# Seed Structure Characteristics to Form Ultrahigh Oil Content in Rapeseed

**DOI:** 10.1371/journal.pone.0062099

**Published:** 2013-04-29

**Authors:** Zhi-Yong Hu, Wei Hua, Liang Zhang, Lin-Bin Deng, Xin-Fa Wang, Gui-Hua Liu, Wan-Jun Hao, Han-Zhong Wang

**Affiliations:** Oil Crops Research Institute of the Chinese Academy of Agricultural Sciences, Key Laboratory of Biology and Genetic Improvement of Oil Crops, Ministry of Agriculture, Wuhan, Hubei, People’s Republic of China; Wuhan University, China

## Abstract

**Background:**

Rapeseed (*Brassica napus* L.) is an important oil crop in the world, and increasing its oil content is a major breeding goal. The studies on seed structure and characteristics of different oil content rapeseed could help us to understand the biological mechanism of lipid accumulation, and be helpful for rapeseed breeding.

**Methodology/Principal Findings:**

Here we report on the seed ultrastructure of an ultrahigh oil content rapeseed line YN171, whose oil content is 64.8%, and compared with other high and low oil content rapeseed lines. The results indicated that the cytoplasms of cotyledon, radicle, and aleuronic cells were completely filled with oil and protein bodies, and YN171 had a high oil body organelle to cell area ratio for all cell types. In the cotyledon cells, oil body organelles comprised 81% of the total cell area in YN171, but only 53 to 58% in three high oil content lines and 33 to 38% in three low oil content lines. The high oil body organelle to cotyledon cell area ratio and the cotyledon ratio in seed were the main reasons for the ultrahigh oil content of YN171. The correlation analysis indicated that oil content is significantly negatively correlated with protein content, but is not correlated with fatty acid composition.

**Conclusions/Significance:**

Our results indicate that the oil content of YN171 could be enhanced by increasing the oil body organelle to cell ratio for some cell types. The oil body organelle to seed ratio significantly highly positively correlates with oil content, and could be used to predict seed oil content. Based on the structural analysis of different oil content rapeseed lines, we estimate the maximum of rapeseed oil content could reach 75%. Our results will help us to screen and identify high oil content lines in rapeseed breeding.

## Introduction

Together with soybean and oil palm, rapeseed is one of the most important oil crops in the world. Because of its high-quality nutritional composition, it is a common source of edible oil. The oil content of the rapeseed at maturity is known to vary among cultivars, and plant breeders have expended a great effort to increase the oil content in the mature seeds of modern oilseed cultivars. According to statistics, the oil content of rapeseed has, at present, increased by 1%, which means the corresponding oil-yield has increased 2.3 to 2.5% [1]. Thus, high-oil-content breeding is a primary improvement strategy for improving the oil yield in oil crops, and it is an essential goal in *B. napus* breeding around the world.

There are numerous species, such as candlenut, sesame, oiticica and ucuhuba, which contain 60 to 76% oil in their seeds [2]. This implies that there is much potentiality to increase the oil content of rapeseed. A scientist of the former Soviet Union forecasted that the oil content of rapeseed might reach 65% [3]. However, at present, the oil content of the main rapeseed cultivars is only 45 to 48% in Canada and 41 to 42% in China and Australia [4]–[5]. In the world, many higher oil content rapeseed lines have been reported [6], such as Major (oil content 50.68%; France), Zephyr (oil content 51.44%; Canada), Zhongyou0361 (oil content 54.72%; China), and HEAR (oil content 54.8%; Canada). In 2009, a Chinese breeder reported the highest oil content of a rapeseed line to date, which was 60% [7]. Our breeding group has recently obtained the ultrahigh oil content rapeseed line YN171, whose oil content is 64.8%, which is 55% greater than the oil content of the main rapeseed cultivars in China.

Plant seeds are complex structures that consist of three major components, each of them with distinct biological roles and fates: (1) the embryo, composed of cotyledon(s), hypocotyl, and radicle; (2) the endosperm, which provides nourishment for the developing embryo; and (3) the seed coat, which surrounds the embryo and the endosperm. It serves as a mechanical protective barrier, and it may be involved in seed dispersal and in the control of germination [8]–[9]. However, in mature rapeseed seeds, the endosperm degenerates and the seed coat enwraps the embryo tightly. Many studies have reported the morphology, structure or development of oil bodies and protein bodies in the seed embryo of rapeseed [10]–[14]. Additionally, some studies reported on the pigment distribution in rapeseed seed coats based on the anatomical structure of the seeds [15]–[16]. Hu et al. [10] found that unusually large oil bodies are highly correlated with a lower oil content in *B. napus*. However, the seed structure and characteristics of ultrahigh oil content rapeseed have not been reported in previous studies.

In this paper we studied the seed structure of ultrahigh oil content rapeseed YN171 at maturity in detail, and compared it with other high oil content rapeseed lines (oil content 45–50%) or low oil content rapeseed lines (oil content 33–35%). In addition, the correlation coefficients of seed oil content, protein content, seed coat ratio, cotyledon ratio, radicle ratio, and 1000-seed weight of 68 *B. napus* lines were investigated. It is very important to analyze the correlation between oil content and other seed traits to determine the main target traits for increasing oil content. The results will help us to screen and identify high oil content lines in rapeseed breeding.

## Results

### Seed Structure of Rapeseed Line YN171 with Ultrahigh Oil Content

Pyramiding breeding over several decades allowed our breeding group to obtain an ultrahigh oil content rapeseed line named YN171, whose oil content is 64.8%. Presently, YN171 has the highest oil content in reported rapeseed lines in the world, and its oil content is 55% greater than the oil content of the main rapeseed cultivars in China. The seed germination of YN171 is normal, and the germinant percent (cotyledon expansion and greening) is about 98%. To investigate the cause and characteristics that lead to this ultrahigh oil content, we determined the structure of mature YN171 seeds by light microscopy and TEM analysis.

**Figure 1:**
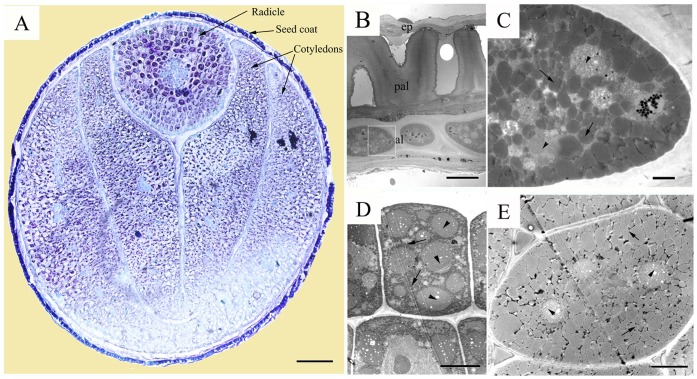
Structure of a mature seed of ultrahigh oil content *B.* *napus* line YN171. (A) Entire seed, showing the seedcoat, radicle and mature cotyledons. Bar = 200 µm. (B) Ultrastructure of the seedcoat, showing the epidermis (ep), palisade (pal) and aleuronic layer (al). (C) Ultrastructure of the aleuronic cell, the local amplification of the white frame part in (B), showing the protein bodies (arrowheads) and oilbodies (arrows). (D) Ultrastructure of the outer radicle cell, showing the protein bodies (arrowheads) and oilbodies (arrows). (E) Ultrastructure of the cotyledon cell, showing the protein bodies (arrowheads) and oilbodies (arrows). Bars are 200 µm in (A), 10 µm in (B), 1 µm in (C) and 5 µm in (D) and (E).

Entire seeds were embedded in paraffin wax according to the method of Hu et al. [10] and 10 µm transverse sections were observed. Light microscopy analysis of entire seeds showed mature seed plumpness, consisting of seed coat and embryo. The seed coat enwrapped the embryo tightly, but not the endosperm (Fig. 1A), and two mature cotyledons enveloped the radicle.

To observe the ultrastructure of YN171 mature seeds, seed coats, cotyledons and radicles were isolated, fixed in 2.5% glutaraldehyde and post fixed in 1% osmium tetroxide solution according to the method of Hu et al. [10]. TEM analysis of YN171 mature seeds revealed that its seed coat consisted of epidermis, palisade and aleuronic layers (Fig. 1B). The thickness of its seed coat is 50 µm on average, and the palisade occupied over half of the seed coat’s breadth. There is only one line of cells in the aleuronic layer, which is about one third of seed coat. It is interesting that both oil bodies and protein bodies were observed in the cells of the aleuronic layer (Fig. 1C), which has not been reported in previous studies. At the same time, oil bodies and protein bodies were also observed in radicle cells and cotyledon cells (Fig. 1D, 1E). In the mature dry seeds, the cytoplasm of the cotyledon cells, radicle cells and aleuronic cells was completely filled with oil bodies and protein bodies. However, the oil body organelles comprised 81% of the total cell area in the cotyledon cells, only 50% in the aleuronic cells and 35% in the radicle cells (Table 1). All oil bodies were spherical in shape, ranging in size from 0.2 to 2.5 µm with the majority of oil bodies <1 µm in diameter, which was consistent with our previous study [10].

**Table 1:** The traits average values of *B. napus* seeds.

Rapeseed	Lipid (%)	Protein (%)	1000-seed weight (g)	Seedcoatratio (%)	Cotyledon ratio (%)	Radicleratio (%)	Oil body organelles ratio (%)		
							Cotyledoncell	Radiclecell	Aleuroniccell
YN171	64.8±1.2	14.5±1.0	4.3±0.1	12.5±0.1	75.3±0.2	12.2±0.2	81±6	35±4	50±4
zy036	49.1±2.6	20.3±1.3	4.5±0.2	14.2±0.7	74.9±1.1	10.9±0.5	58±5	31±5	38±5
6F313	47.3±2.4	21.8±2.2	5.2±0.1	14.8±0.2	74.6±0.2	10.6±0.2	53±3	44±5	33±4
09–88	47.0±2.1	23.0±1.5	5.1±0.2	13.4±0.5	73.3±0.2	13.3±0.6	55±5	36±4	27±4
51070	34.9±2.6	29.0±2.1	3.6±0.2	23.7±1.2	59.5±0.8	16.8±0.4	38±4	29±6	20±5
7ml177-3	33.7±1.8	29.4±1.2	4.5±0.2	15.7±0.6	72.6±0.6	11.7±0.2	35±3	23±5	26±4
9Q629-3	33.2±1.6	25.6±1.3	5.1±0.1	18.3±0.5	70.7±0.1	11.0±0.4	33±3	26±4	28±5

Mean values are given in percentage of seed dry weight with standard deviations. *n*≧3.

### Comparative Analysis of Mature Seed Morphology and Structure

To compare the mature seed morphology and structure among different rapeseed lines, the ultrahigh oil content line YN171 (oil content of 64.8), three high oil content lines ZY036, 6F313 and 09–88 (oil content of 49.1, 47.3 and 47.0, respectively) and three low oil content lines, 51070, 7ml177-3 and 9Q629-3 (oil content of 34.9, 33.7 and 33.2, respectively) from the 68 rapeseed lines were chosen (Table 1). An inverse relationship between oil and protein accumulation existed, but there was no obvious correlation between 1000-seed weight and oil accumulation in the different lines.

**Figure 2:**
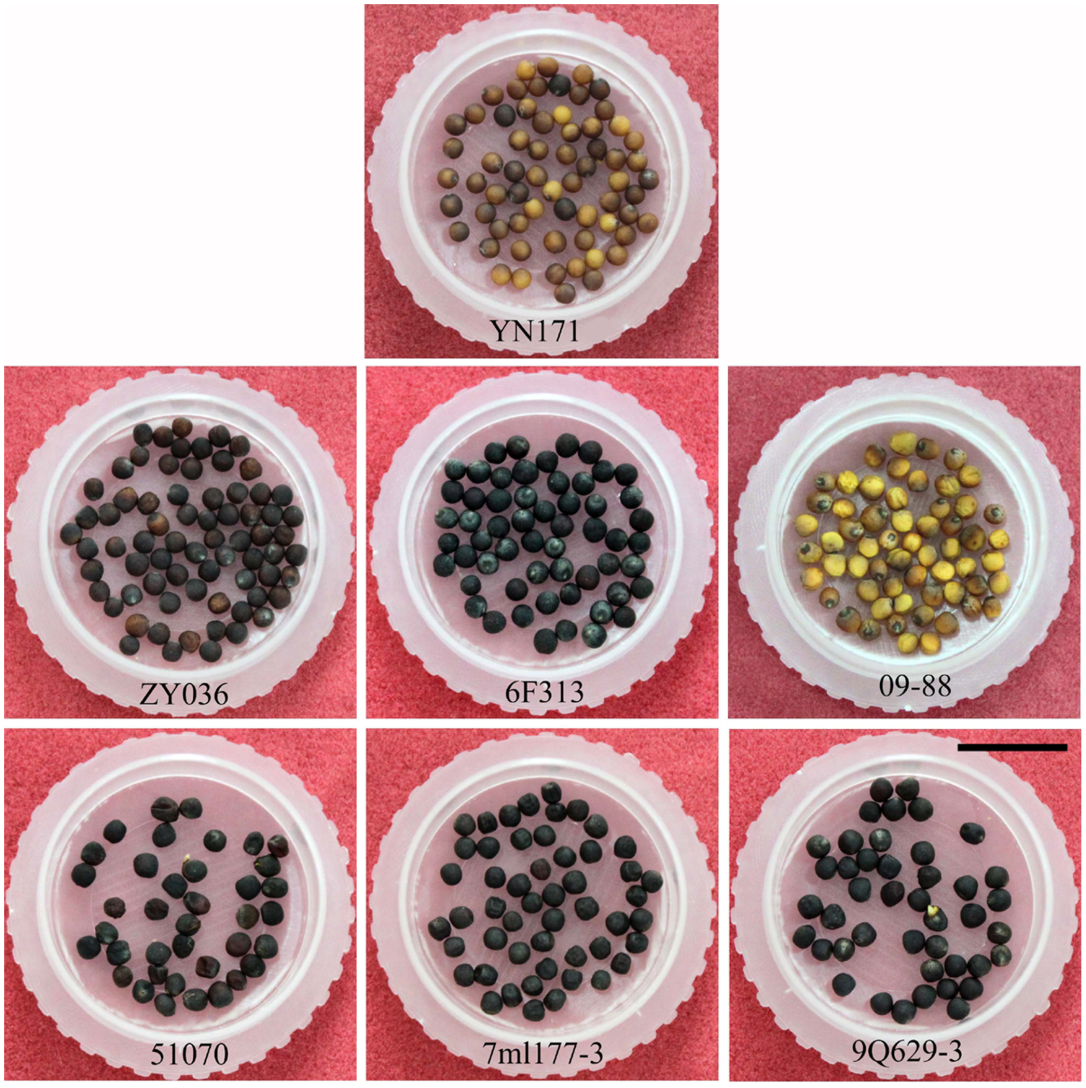
Seed morphology of 7 rapeseed lines with different oil content. The upper is the ultrahigh oil content line YN171, the middle is three high oil content lines ZY036, 6F313 and 09–88, the nether is three low oil content lines 51070, 7ml177-3 and 9Q629-3. Bar is 1 cm.

The seed coat of YN171 was yellow-brown, and it was black in the three low oil content lines (Fig. 2). However, in the three high oil content lines there were various seed coat colors, including yellow-brown, black and yellow.

**Figure 3:**
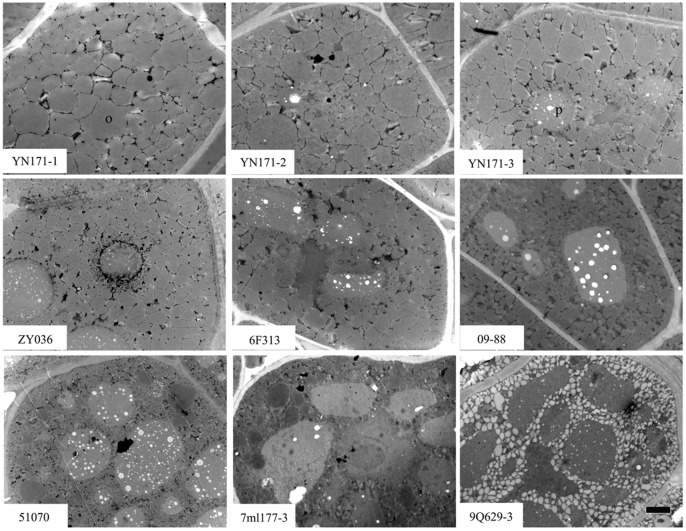
TEM analysis of 7 lines different oil content rapeseed mature cotyledons. The upper is the ultrahigh oil content line YN171 with three observations (YN171-1, YN171-2 and YN171-3), the middle is three high oil content lines ZY036, 6F313 and 09–88, the nether is three low oil content lines 51070, 7ml177-3 and 9Q629-3. o, oil body; p, protein body; Bar is 2 µm.

TEM analysis of mature cotyledons revealed oil body organelles comprised 81% of the total cell area in the ultrahigh oil content line, 53 to 58% in the three high oil content lines, and only 33 to 38% in the three low oil content lines (Table 1, Fig. 3). On the contrary, there were only some sporadic small protein bodies observed in the ultrahigh oil content line, and the most abundant and biggest protein bodies appeared in the three low oil content lines. The number of protein bodies in the high oil content lines was between those of ultrahigh and low oil content lines.

**Figure 4:**
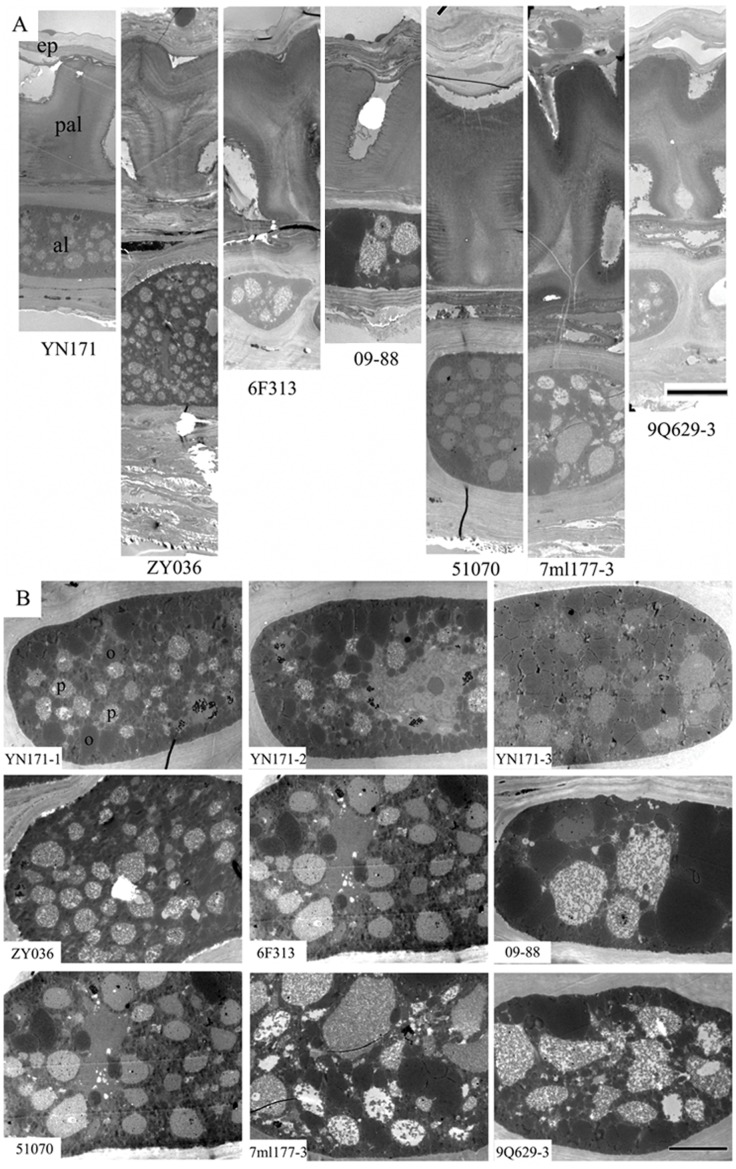
TEM analysis of 7 lines different oil content rapeseed seedcoats. (A) Ultrastructure of seedcoat, showing the epidermis (ep), palisade (pal) and aleuronic layer (al). From left to right, YN171, ZY036, 6F313, 09–88, 51070, 7ml177-3 and 9Q629-3. (B) Ultrastructure of aleuronic cell in rapeseed seedcoat, showing the oilbodies (o) and protein bodies (p). The upper is the ultrahigh oil content line YN171 with three observations (YN171-1, YN171-2 and YN171-3), the middle is three high oil content lines ZY036, 6F313 and 09–88, the nether is three low oil content lines 51070, 7ml177-3 and 9Q629-3. Bars are 10 µm in (A) and 5 µm in (B).

In the seven rapeseed lines with different oil contents, TEM analysis of seed coats revealed various thicknesses (Fig. 4A). The seed coat was only about 50 µm thick in the ultrahigh oil content line, 60 to 110 µm thick in the three high oil content lines, and 75 to 110 µm thick in the three low oil content lines. Oil bodies and protein bodies were observed in the aleuronic cells of all seven rapeseed lines (Fig. 4B). The distribution of oil bodies and protein bodies in the aleuronic cells was similar with that in the cotyledons. There were more oil bodies and less protein bodies in the ultrahigh oil content line than in the high oil content lines and low oil content lines. Oil body organelles comprised 50% of the total aleuronic cell area in the ultrahigh oil content line, 27 to 38% in the three high oil content lines, and only 20 to 28% in the three low oil content lines (Table 1).

**Figure 5:**
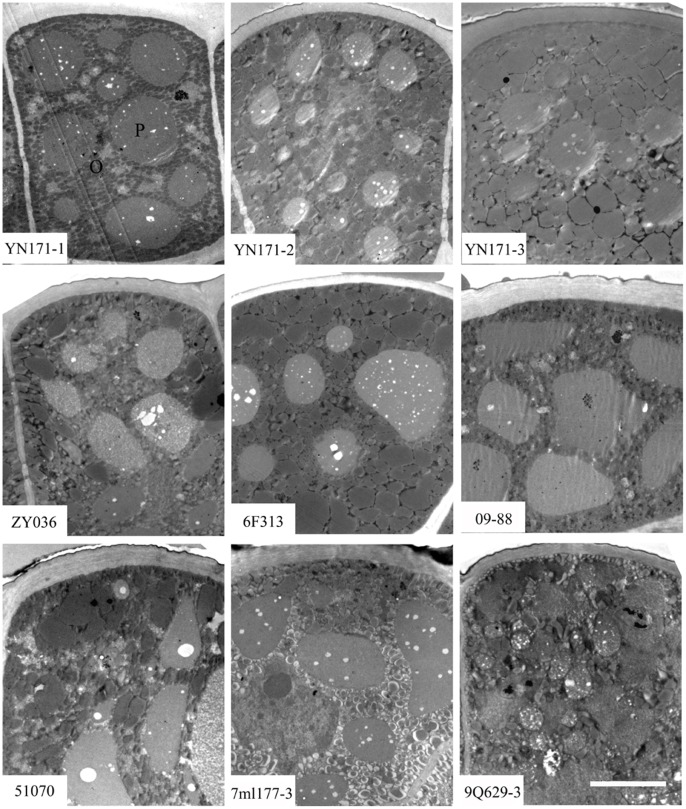
TEM analysis of 7 lines different oil content rapeseed radicles. The upper is the ultrahigh oil content line YN171 with three observations (YN171-1, YN171-2 and YN171-3), the middle is three high oil content lines ZY036, 6F313 and 09–88, the nether is three low oil content lines 51070, 7ml177-3 and 9Q629-3. o, oil body; p, protein body; Bar is 5 µm.

TEM analysis of radicles revealed oil bodies and protein bodies were also observed in all seven rapeseed lines with different oil contents (Fig. 5). There were no obvious differences in the distribution of oil bodies and protein bodies in radicles between the ultrahigh oil content line and the high oil content lines. There were more oil bodies and less protein bodies in the ultrahigh oil content line and the high oil content lines than in the low oil content lines. Oil body organelles comprised 35% of the total cell area in the ultrahigh oil content line, 31 to 44% in the three high oil content lines and only 23 to 29% in the three low oil content lines (Table 1).

### Comparative Analysis of Fatty Acid Composition in Different Oil Content Rapeseed Lines

**Figure 6:**
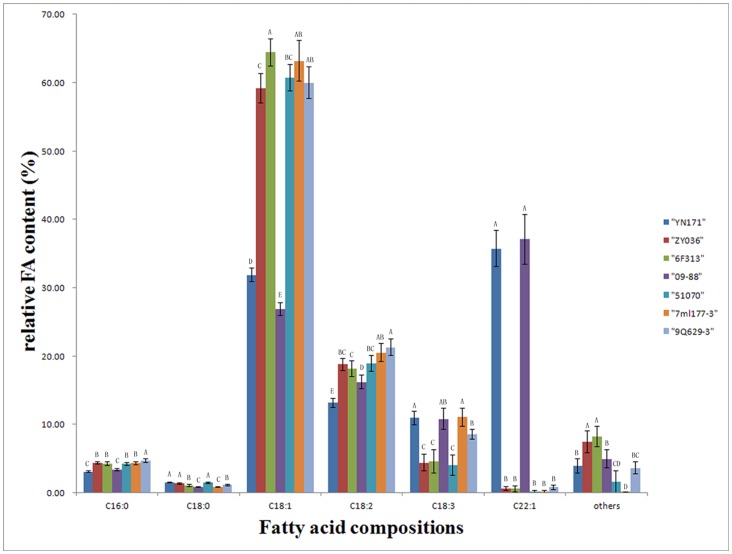
Fatty acid profiles of *B.* *napus* seeds.

Lipids are generally stored as triacylglycerols in oil bodies. Although there are quantitative differences in the accumulation of storage reserves in seeds, it was not clear whether this would also qualitatively affect the fatty acid profiles of triacylglycerols in these seeds. Therefore, fatty acid compositions in these seven rapeseed lines were measured. As shown in Fig. 6, the results suggested there were two main types with either a high or a low C22∶1 (erucic acid) content. The C22∶1 content of the ultrahigh oil content line, YN171, and one high oil content line, 09–88, were over 35%, but the C22∶1 content was less than 1% in the other five lines with either high or low oil contents. After C22∶1, C18∶1 (oleic acid) was the main fatty acid component. Its contents were about 30% in the two lines with high C22∶1 content and about 60% in the remaining. The others fatty acids did not show any statistical differences. Our results indicated that the oil content was not correlated with fatty acid composition. Breeding varieties with high oleic acid for the food industry or high erucic acid for non-food applications in oilseed are two major objectives of *B. napus* breeding programs [17].

### Analysis of Correlations between Oil Content with Other Seed Traits

The correlation analyses of six mature seed traits, including oil content, protein content, seed coat ratio, cotyledon ratio, radicle ratio and 1000-seed weight of 68 *B. napus* lines were performed. The correlation coefficients of all six mature seed traits were analyzed, and the results are provided in Table 2.

**Table 2:** Correlation coefficients of seed oil content, protein content, seedcoat ratio, cotyledon ratio, radicle ratio, and 1000-seed weight of 68 *B. napus* lines.

	Oil content	Protein content	Seedcoat ratio	Cotyledon ratio	Radicle ratio	1000-seed weight
Oil content	1					
Protein content	−0.74**	1				
Seedcoat ratio	−0.54**	0.11	1			
Cotyledon ratio	0.43**	−0.08	−0.90**	1		
Radicle ratio	−0.07	−0.00	0.35**	−0.71**	1	
1000-seed weight	−0.11	0.19	−0.21	0.43**	−0.60**	1

* Significant at p = 0.05,

** significant at p = 0.01.

A significant negative correlation between the oil and protein contents and the seed coat ratio existed, with correlation coefficients of −0.74 (p<0.01) and −0.54 (p<0.01), respectively. On the contrary, a significant positive correlation was observed between the oil content and the cotyledon ratio, with a correlation coefficient of 0.43 (p<0.01).

The seed coat ratio had a significant negative correlation with the cotyledon ratio, and the coefficient was −0.90 (p<0.01). However, there was a significant positive correlation between the seed coat ratio and the radicle ratio, with a coefficient of 0.35 (p<0.01). The cotyledon ratio had a significant negative correlation with the radicle ratio, and the coefficients was −0.71 (p<0.01). However, there was a significant positive correlation between the cotyledon ratio and the 1000-seed weight, with a coefficient of 0.43 (p<0.01). There was a significant negative correlation between the radicle ratio and the 1000-seed weight, with a correlation coefficient of −0.60 (p<0.01).

### Analysis of Correlation between Oil Content and Oil Body Organelle to Seed Ratio

Rapeseed seed is consisted of seed coat, cotyledon and radicle. The oil body organelle is accumulated in aleurone cells of seed coat, cotyledon cells and radicle cells. The oil body organelle to cell area ratios for aleurone cells, cotyledon cells, and radicle cells could be measured by TEM analysis (Table 1). The aleurone (about 1/3 of seedcoat), cotyledon and radicle ratios to seed also are measured in Table 1. Thus the oil body organelle to seed ratio could be calculated. In order to analyze the relationship between oil content with the oil body organelle to seed ratio, an expression was used to calculate the oil body organelle to seed ratio as following: the oil body organelle to seed ratio = (Cotyledon ratio × Oil body organelles ratio in cotyledon cell+Radicle ratio × Oil body organelles ratio in radicle cell +1/3 × Seedcoat ratio × Oil body organelles ratio in aleuronic cell). According to the data in table 1, the oil body organelle to seed ratio of seven rapeseed lines was 67.3, 48.6, 45.8, 46.3, 29.1, 29.5 and 27.9 respectively. Correspondingly, the oil content of these seven rapeseed lines was 64.8, 49.1, 47.3, 47.0, 34.9, 33.7 and 33.2 respectively. The correlation analysis indicated that oil content is highly significantly positively correlated with oil body organelle to seed ratio, and the correlation coefficient is 0.99 (p<0.01). That means the oil body organelle to seed ratio is highly close to the oil content. In other words, the oil body organelle to seed ratio could be used to predict seed oil content.

## Discussion

### The High Oil Body Organelles Ratio to Cotyledon Cells Contributes to the Ultrahigh Oil Content of YN171

The seeds of many plant species, including rapeseed, accumulate lipids to supply the energy requirements for germination and seedling growth. Such lipids are generally stored as triacylglycerols in small, spherical and discrete intracellular organelles called oil bodies [10], [18]–[20]. Thus, the greater the number of oil bodies, the higher the oil content of a seed.

In rapeseed, as in sunflowers, all cell types in the embryo form oil bodies, including the radicle cells [21]. In addition, we observed oil bodies in aleuronic cells of the seed coat in rapeseed, which has not been previously reported. In this study, oil bodies were found to have accumulated in cotyledon cells, radicle cells, and aleuronic cells, and the oil body to organelle area ratio is about 81%, 35% and 50%, respectively. The cotyledon ratio, radicle ratio and seed coat ratio of YN171 are 75.3%, 12.2% and 12.5%, respectively (Table 1). Thus, about 80 to 90% of seed oil comes from the cotyledon. In the three high oil content lines, the oil body organelle to cotyledon cell ratio is only 53 to 58%, which is lower than in YN171. Thus, the high oil body organelle to cotyledon cell ratio contributes to the ultrahigh oil content of YN171.

### Oil Content is Significantly Negatively Correlated with Protein Content

Lipids and protein are major storage reserves in mature *Brassica* seeds [22]. In mature seeds, lipids are stored in oil bodies and proteins in protein bodies [12]. An inverse relationship between oil and protein accumulation in seeds has been reported for some plant species, including rapeseed [10], [23]–[24]. Our results further corroborated that seed oil content was significantly negatively correlated with protein content, with a correlation coefficient of −0.74. The protein content is only 14.5% in the ultrahigh oil content line YN171, which is the lowest protein content in the 68 rapeseed lines studied.

### YN171 is an Excellent Breeding Material for Genetic Improvement of the Oil Content

The edible and processing quality of rapeseed oil is mainly determined by the fatty acid composition of the triglycerol lipids in its seeds [25]. Our ultrahigh oil content line, YN171, not only contained high oleic acid but also contained high erucic acid. Generally, oleic acid is optimal fatty acids for human health and nutrition and erucic acid is unsuitable for edible. Oil low in erucic acid contains a nearly optimal balance of fatty acids for human health and nutrition, and it has been widely used in the food industry [17]. Thus, YN171 is not suitable for use directly in production. However, our results showed that oil content was not correlated with fatty acid composition. High erucic acid content could be reduced through genetic improvement in rapeseed breeding.

On the other hand, erucic acid is one of the main fatty acids in rapeseed oil. High erucic acid rapeseed has the potential to be used in the oleo-chemical industry in the production of items such as high temperature lubricants, nylon, plastics, slip and coating agents, soaps, painting inks and surfactants [26]. It is also an ideal raw material for biodiesel production [27]. Genetic improvement of oilseeds has the objective of increasing oil yields with a uniform fatty acid composition for nutritional, pharmaceutical and industrial purposes [28]. Thus the ultrahigh oil content line YN171 provided us an excellent material for high oil content rapeseed breeding.

### Seed Structure Basis of High Oil Content Breeding in Rapeseed

On the one hand, oil bodies were mainly accumulated in embryo organ especially in cotyledon of rapeseed seed. The bigger the cotyledon ratio in seed is, the higher the seed oil content is. That’s means the seed coat should be thin to reach high oil content. In general, yellow rapeseed seeds have a thinner seed coat than the black seeds lines. However, not all high oil content rapeseed lines have yellow seeds, some black rapeseed lines, such as 6F313 in this study, are also high in oil content. Tang et al. [29] reported that the oil content of the seed coat of yellow seeds is 3% higher than that of the brown seeds of the same genetic background, and that there were no obvious differences between yellow and brown seeds in the embryonic oil content. The seed coat ratio of yellow seeds is 4.2% lower than that of brown seeds [29]. Research has indicated that the embryonic oil content does not appear different between yellow and black seeds; however, yellow seeds have a higher total oil content because their embryo ratio and seed coat oil content have increased [29]–[30]. Our results are consistent with previous studies. The correlation analysis of six mature seed traits revealed that the seed oil content is significantly negatively correlated with the seed coat ratio and significantly positively correlated with the cotyledon ratio.

On the other hand, the cytoplasms of cotyledon, radicle, and aleuronic cells were completely filled with oil and protein bodies. The less the protein body is, the more the oil body is. The correlation analysis also revealed that the seed oil content is significantly negatively correlated with the protein content. Therefore, it is hypothesized that to obtain an ultrahigh oil content rapeseed line the yellow or yellow-brown seed coat color is optimal, because its seed coat ratio is low, cotyledon ratio is high and it has high embryonic and seedcoat oil contents. In addition, there should be a high oil body organelle to cell area ratio in all cell types, including cotyledon cells, radicle cells, and aleurone cells.

### The Maximum Oil Content in Rapeseed might be Increased to 75%

Increasing seed oil content is a major goal for rapeseed breeding. In our ultrahigh line, the oil content is 64.8%, and the oil body organelle to cell area ratios for cotyledon cells, radicle cells, and aleurone cells are 81%, 35% and 50%, respectively. Observed by TEM analysis, only some sporadic small protein bodies appeared in parts of the cotyledon cells, and the other cotyledon cells were completely filled with oil bodies. This led to a high oil body organelle to cell area ratio of 98%. Additionally, the oil body organelle to cell area ratios in the radicle cells of the two high oil content lines, 6F313 and 09–88, are 44% and 36%, respectively, which is higher than that of YN171 (Table 1). This implied that the oil body organelle to cell area ratio in some cells has the potential to be increased. If the oil body organelle to cell area ratio in cotyledon cells and radicle cells was increased to 90% and 44%, respectively, and maintained the 50% in aleurone cells, the oil content of rapeseed might reach 75% (Predict according to the oil body organelle to seed ratio).

On the other hand, the total content of lipid and protein has reached 79.3% in YN171, which means only about 20.7% of the cell components were necessary for maintaining the structure and frame of rapeseed seed cells. That’s means the 75% oil content in rapeseed could be achieved in theory. Several decades ago, a former Soviet Union scientist forecasted rapeseed oil content of 65% [3] that has come true. Now we optimistically forecast a 75% oil content in rapeseed.

## Materials and Methods

### Plant Material

*B. napus* lines exhibit a wide range of oil contents in the mature seed. Sixty-eight mature rapeseed seeds with oil contents from 33.2% to 64.8% were collected in China (see Table S1 in Supporting Information). All plant materials were planted in a field located in Wuhan and Yunnan, China. Seeds of all lines were collected after maturation in 2012.

### Characterization of Mature Seed Traits of Rapeseed

Lipid and protein contents were analyzed according to the method of Hu et al. [10]. The lipid contents, including fatty acid composition, and proteins contents were determined using a Foss NIRSystems 5000 (Foss NIRSystems Inc., Silver Spring, MD, USA). At the same time, the lipid contents of the ultrahigh oil content rapeseed line YN171 was analyzed by the Soxhlet extraction method by the Xi’an Grease Food and Feed Quality Supervision and Testing Center of the State Administration of Grain in China.

To determine the seed coat ratio, cotyledon ratio and radicle ratio, the seed coat, cotyledon and radicle were isolated from dry seeds, and 10 seeds, with three replicates, were used in the experiment. To determine the 1000-seed weight, 10 seeds, with three replicates, were used in the experiment. The traits averaged using the values of 68 mature rapeseed seeds are shown in Table S1. To determine the oil body organelle to cell ratios for cotyledon cells, radicle cells and aleuronic cells, the areas of oil body and cells were measured, with ten replicates, using Image Pro Express software.

### Light Microscopy

Mature rapeseed seeds were imbibed for 2 to 3 h and fixed overnight in a solution containing 10% formaldehyde, 50% ethanol, and 5% acetic acid in water. After dehydration using an ethanol series (50%, 70%, 95%, and 100%), the seeds were cleared twice with xylene for 2 h. The seeds were infiltrated and subsequently embedded in paraffin wax according to the method of Hu et al. [10]. Sections were obtained using a Leica RM 2016 microtome (Leica, Nanterre Cedex, France) and stained with 0.1% toluidine blue for 5 min. The observations were made with a light fluorescence microscopy (IX-71; Olympus, Tokyo, Japan). Images were acquired using a DP 71 CCD camera (Olympus, Tokyo, Japan).

### Transmission Electron Microscopy (TEM)

For transmission electron microscope analysis, seed coats, cotyledons and radicles were isolated from dry seeds, fixed immediately in 2.5% glutaraldehyde, and postfixed with a 1% osmium tetroxide solution according to the method of Hu et al. [10]. After dehydration using an acetone series (30%, 50%, 75%, 90%, 100%), the tissues were infiltrated and subsequently embedded in EPON-812 epoxy resin. Ultrathin sections were prepared using a diamond knife on an UC6 Ultratome (Leica, Austria) and stained with uranyl acetate and lead citrate. The stained sections were examined on an H-7650 transmission electron microscope (Jeol, Tokyo, Japan).

### Data Analysis

Correlation coefficients between traits were analyzed with Statistical Analysis System (SAS) V8 (SAS Institute Inc., Cary, NC, USA).

## Supporting Information

Table S1 The trait average values of 68 lines mature rapeseed seeds.
(XLSX)
